# Bioinformatics Strategy for 16s and 23s rRNA Metabarcoding Data

**DOI:** 10.3390/biotech15020042

**Published:** 2026-06-08

**Authors:** Rita Domingues, José C. M. Pires

**Affiliations:** LEPABE (Laboratory for Process Engineering, Environment, Biotechnology and Energy), ALiCE (Associate Laboratory in Chemical Engineering), Faculty of Engineering, University of Porto, Rua Dr. Roberto Frias, 4200-465 Porto, Portugal; domingues.anarita@gmail.com

**Keywords:** bioinformatics, metagenomics, 16S rRNA, 23S rRNA, OTU clustering, VSEARCH

## Abstract

Understanding biological communities is essential for elucidating ecosystem structure and function. Metabarcoding based on ribosomal RNA (rRNA) genes, particularly 16S and 23S, is widely used to characterise bacterial and microalgal communities. However, analysing high-throughput sequencing data generated by platforms such as the Illumina MiSeq remains challenging due to fragmented bioinformatics tools, complex parameterisation, and limited accessibility for non-specialist users. In this study, a comprehensive and user-friendly bioinformatics pipeline is proposed for the analysis of 16S and 23S paired-end metabarcoding data. The workflow integrates all critical processing steps, including read merging, primer and adapter trimming, quality filtering, dereplication, chimaera removal, and clustering into Operational Taxonomic Units (OTUs). Taxonomic assignment is performed using curated reference databases, namely EZBioCloud for bacterial communities and µgreen for microalgae. The pipeline was developed in Python 3.11 and incorporates validated tools such as VSEARCH and Cutadapt, ensuring robustness and computational efficiency. Additionally, modules for alpha and beta diversity analysis are included to support comprehensive ecological interpretation. The main novelty of this work lies in providing a unified, GUI-based framework that enables the standardised processing of dual-marker (16S/23S) metabarcoding data within a single environment. In its current implementation, SOMBA supports the analysis of each marker through separate but harmonised workflows, ensuring consistency in parameterisation, processing steps, and output structure. This approach provides an accessible and standardised solution that bridges the gap between raw sequencing data and reliable biological insights, supporting applications in environmental microbiology and biotechnology.

## 1. Introduction

The intricate web of life within any defined spatial unit, collectively termed a biological community, is a cornerstone of ecological and biological research. Understanding the constituent organisms, their roles, and their interactions provides fundamental insights into ecosystem functioning, evolutionary processes, and environmental health [1,2]. Metagenomics has emerged as a transformative discipline that enables the study of entire microbial communities directly from environmental samples, bypassing the need for cultivation [3]. Unlike traditional genomics, which focuses on the genetic material of a single organism, metagenomics delves into the collective genomes of multiple organisms within a given sample, offering an unparalleled resolution for characterising taxonomic profiles and functional potentials. The versatility of metagenomic approaches has led to their extensive application across diverse fields, ranging from medical diagnostics and industrial biotechnology to environmental monitoring and agricultural sciences [4,5].

A typical metagenomic study follows a multi-stage roadmap: meticulous sample collection, DNA extraction, targeted gene amplification, library preparation, and high-throughput sequencing. The ultimate, and often most complex, stage involves sophisticated bioinformatics analysis to interpret the vast quantities of data generated [4]. The selection of appropriate methods at each step is paramount, as unsuitable choices can introduce biases and lead to erroneous conclusions [6]. Critical decisions include choosing between targeted and non-targeted sequencing approaches and selecting the specific sequencing platform, all of which must align with the overarching research objectives [4,5,7].

Among the various strategies for generating taxonomic profiles, the sequencing of amplicons derived from phylogenetic marker genes (a targeted method) is widely favoured. Ribosomal RNA (rRNA) genes are considered ideal phylogenetic markers due to their universal presence across all cellular life forms and their mosaic structure, which features both highly conserved regions and hypervariable domains [8,9,10]. The conserved regions serve as robust binding sites for universal PCR primers, facilitating amplification, while the hypervariable regions exhibit sufficient sequence divergence to enable taxonomic discrimination. In prokaryotes (Bacteria and Archaea), the small ribosomal subunit contains the 16S rRNA gene, whereas the large subunit contains the 5S and 23S rRNA genes. Eukaryotes possess 18S rRNA in the small subunit and 5S, 5.8S, and 25S/28S rRNAs in the large subunit. The 16S rRNA gene has historically been the primary focus for bacterial and archaeal community profiling due to its manageable length and extensive reference databases, making it instrumental in microbial ecology and systematics. Concurrently, the 23S rRNA gene, particularly from chloroplasts, is increasingly recognised as a robust taxonomic marker for microalgae, facilitating the study of these vital photosynthetic organisms [11,12,13].

Advances in sequencing technologies have dramatically enhanced the throughput and efficiency of metagenomic studies. Next-Generation Sequencing (NGS) platforms, characterised by massively parallel sequencing capabilities, offer ultra-high throughput, scalability, and accelerated data generation rates [14,15]. Illumina technology (Illumina, Inc.) has become a dominant force in this arena, largely owing to its cost-effectiveness, high yields, and high-quality sequencing. The robust capacity of these high-throughput sequencers now enables researchers to investigate subtle shifts in biodiversity across various environments and monitor ecological dynamics over extended periods [16].

However, the sheer volume of sequencing data produced by NGS platforms presents significant bioinformatics challenges. Differentiating genuine biological signals from sequencing errors and artefactual sequences is critical for achieving high-resolution taxonomic profiles. Biological limitations, such as intragenic variations and differential gene evolution rates across taxa, further complicate analysis, as do technical limitations inherent in the sequencing process itself [17]. Consequently, simply enumerating every unique sequence is often impractical and can lead to an overestimation of diversity. Instead, Operational Taxonomic Units (OTUs) serve as a fundamental concept in taxonomic profiling. Reads are clustered into OTUs based on a predefined sequence similarity threshold, typically 97% for prokaryotes and 99% for eukaryotes, which is generally considered to correspond to species-level resolution [17,18].

The OTU-based approach offers flexibility, allowing researchers to adjust clustering stringency to align with specific project requirements and biological questions. Nevertheless, the chosen clustering methodology significantly impacts the precision and accuracy of OTU assignments. Three primary methodologies for OTU clustering have been developed: closed-reference, de novo, and open-reference clustering [17,19]. Closed-reference clustering aligns sequences against a well-established reference database, grouping reads that align well to the same reference sequence into a single OTU. Reads that do not match any reference sequence are typically discarded. This approach is computationally efficient but may overlook novel or rare taxa not present in the reference database. Conversely, de novo clustering operates independently of existing databases and performs pairwise comparisons among all experimental reads. While this method is more adept at identifying novel and rare taxa, it is computationally intensive, requiring substantial memory and processing time for large datasets. Open-reference clustering is a hybrid approach: it first performs closed-reference clustering and then applies de novo clustering to reads that fail to match any reference sequence. In theory, this method aims to combine the efficiency of closed-reference clustering with the comprehensiveness of de novo clustering. However, the effectiveness of hybrid approaches can vary, and careful consideration is required. The optimal choice of clustering strategy ultimately depends on the nature of the sequencing data, the research objectives, and the completeness and robustness of the available reference databases.

The growing array of bioinformatics software packages and the specialised programming skills required to utilise them effectively can be intimidating for researchers embarking on large-scale sequencing data analysis. A limited body of literature provides comprehensive comparisons of the advantages and disadvantages of various available software tools. Furthermore, despite sharing the same overarching goal, different bioinformatics pipelines can introduce biases due to variations in their constituent steps or parameter choices. Although some processing steps are universally recognised as mandatory for generating reliable results, the specific implementation and parameter optimisation often diverge between pipelines [20,21]. Given that many pipelines rely on heuristic methods, it is not uncommon for them to yield different results even when applied to identical datasets. Therefore, a thorough and transparent definition of the bioinformatics approach, including a detailed account of parameter optimisation strategies, is essential for ensuring the robustness and reproducibility of metagenomic analyses.

VSEARCH [22] has gained recognition as a highly efficient tool for characterising taxonomic profiles, particularly well-suited for Illumina sequencing data [20,22]. As an open-source, multithreaded 64-bit tool designed for metagenomics, VSEARCH serves as a powerful alternative to proprietary software such as USEARCH. Its open-source nature allows for transparency and community-driven development, a significant advantage over tools with undisclosed algorithms [22]. Prior studies have demonstrated that VSEARCH performs comparably to or even surpasses USEARCH in terms of accuracy [17,22].

This paper presents a detailed computational methodology, embodied in a structured bioinformatics pipeline, designed to streamline the analysis of microbial and microalgal metagenomic data for end users. The approach focuses on developing a comprehensive pipeline with methodically optimised settings tailored for 16S and 23S rRNA metabarcoding analysis, emphasising open-source software and a user-friendly interface.

## 2. Materials and Methods

This section outlines the types of sequencing data that the bioinformatics strategy is designed to process, the reference databases utilised for taxonomic assignment, and the software environment in which the pipeline is developed and executed. The descriptions herein focus on the methodological considerations rather than specific experimental contexts or results.

### 2.1. Sequencing Data Considerations

The presented bioinformatics pipeline is designed for the comprehensive analysis of high-throughput sequencing data, specifically paired-end reads generated by the Illumina MiSeq™ platform. The methodology is optimised for phylogenetic markers, including the V3-V4 domain of the 16S rRNA gene, commonly used for bacterial and archaeal community profiling, and the V5 domain of the 23S rRNA gene, which is increasingly applied for microalgal community characterisation. Input data are expected to be in standard FASTQ format, with forward and reverse reads already demultiplexed, meaning sequences originating from different samples have been separated into individual files. The paired-end nature of the data is crucial for subsequent merging steps that enhance read quality and length, improving downstream analysis accuracy. The pipeline’s architecture allows for flexible adaptation to various sample types, provided the sequencing data adhere to these general specifications.

### 2.2. Reference Databases

The accuracy of taxonomic assignment in metagenomic pipelines depends fundamentally on the quality, comprehensiveness, and appropriate formatting of the reference databases used. For the methodology described, two publicly accessible and robust databases are utilised, tailored to the specific phylogenetic markers targeted:

- EZBioCloud [23,24]: This database serves as the primary reference for the classification of 16S rRNA gene sequences. EZBioCloud is renowned for its comprehensive collection of 16S rRNA gene sequences from formally described and validated bacterial and archaeal species, curated with a strong emphasis on taxonomic accuracy. Its rigorous quality control and taxonomic hierarchy make it a highly reliable resource for microbial community analysis.
- µgreen [13]: Dedicated to microalgae, the µgreen database provides reference sequences for the 23S rRNA gene, among other markers. This specialised database is crucial for the accurate identification and classification of microalgal taxa, which are often underrepresented or less thoroughly classified in broader microbial databases. The µgreen database is designed to support detailed phylogenetic analyses of diverse microalgal communities.

A critical consideration for seamless integration with the pipeline’s taxonomic classification module is the specific format of the reference sequences within these databases. To ensure compatibility and enable precise taxonomic assignment across multiple hierarchical levels, the FASTA-formatted database files must adhere to a standardised header structure. Each reference sequence header must contain taxonomic labels in the following explicit format: >code;tax= p:phylum_name,c:class_name,o:order_name,f:family_name,g:genus_name,s:species_name.

This structured format allows the classification algorithm to parse and assign taxonomy consistently and accurately at seven distinct levels: phylum, class, order, family, genus, and species. Any reference database intended for use with this methodology must undergo a preliminary formatting step to conform to this specified syntax, ensuring that all necessary taxonomic information is readily available and correctly interpreted by the pipeline.

### 2.3. Software Environment

The bioinformatics pipeline presented in this work, internally referred to as SOftware for MetaBarcoding Analysis (SOMBA, v1.0), is developed in Python 3.11. Python was chosen for its versatility, extensive libraries, and readability, which facilitate robust script development and maintenance. The core functionalities of the pipeline, particularly for sequence processing and manipulation, rely on powerful external dependencies:

- VSEARCH 2.22.1 [22]: This open-source, multithreaded 64-bit tool is central to the metagenomic analysis workflow. VSEARCH is selected for its high performance, accuracy, and efficiency in handling large sequence datasets, making it an excellent choice for tasks such as read merging, quality filtering, dereplication, chimaera detection, and OTU clustering. Its open-source nature ensures transparency and allows for its free use in academic and research settings.
- Cutadapt 4.4 [25]: Cutadapt is used to precisely identify and remove adapter sequences and primers from sequencing reads. It is a highly flexible tool essential for cleaning raw sequencing data, ensuring that only biologically relevant sequences proceed to downstream analysis.
- scikit-bio™ [7,26,27]: This open-source bioinformatics package in Python is utilised for the calculation of various alpha and beta diversity indices. The scikit-bio tool provides a robust, well-validated framework for ecological and phylogenetic analyses, ensuring the accuracy of the diversity metrics computed within the pipeline. The reference for this package is available at https://doi.org/10.5281/zenodo.8209901.

To enhance accessibility and ease deployment across different computational environments, the entire Python-based algorithm can be compiled into a standalone executable. This functionality is achieved using *PyInstaller*, which bundles the Python interpreter and all necessary dependencies into a single package. This allows SOMBA to run on any operating system without requiring the user to install Python or its specific dependencies separately, thereby significantly lowering the barrier to entry for researchers without extensive programming expertise. The graphical user interface, developed with Python’s Tkinter package, further enhances the software’s user-friendliness, enabling intuitive parameter control and workflow management.

The SOMBA software and the corresponding User Guide are provided as Supplementary Material accompanying this manuscript. The User Guide includes detailed information regarding the required computational environment and all dependencies necessary for proper execution. SOMBA is designed to run on Windows operating systems, as it was developed using tools and dependencies compatible with this environment.

## 3. Bioinformatics Pipeline

The methodology adopted to characterise the taxonomic profile of samples involves a structured, sequential bioinformatics pipeline. This section provides a detailed breakdown of each processing step, the underlying rationale, and the specific software commands and parameters employed. The design emphasises robustness, efficiency, and the ability to handle large volumes of paired-end sequencing data from Illumina MiSeq platforms.

### 3.1. Overview of the SOftware for MetaBarcoding Analysis

SOMBA is a comprehensive, Python-based pipeline designed to process 16S and 23S rRNA metabarcoding data from raw sequencing reads through to annotated taxonomic profiles and diversity metrics. The pipeline integrates external, open-source bioinformatics tools, primarily VSEARCH and Cutadapt, within a cohesive framework. A key feature of SOMBA is its compilation into a standalone executable, facilitating cross-platform compatibility and ease of use. A graphical user interface (GUI), conceptually similar to a modular workflow diagram, allows users to intuitively manage and optimise the various parameters at each stage of the analysis.

**Figure 1:**
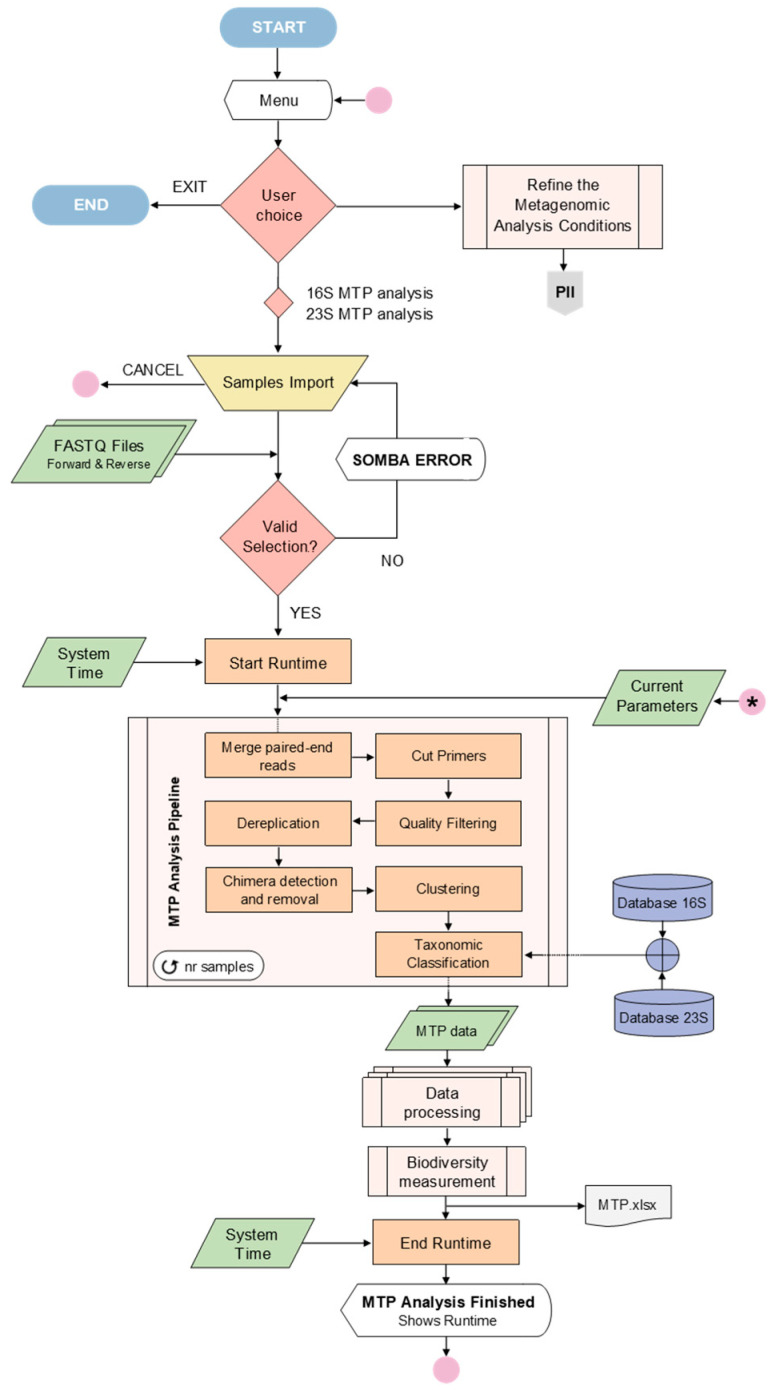

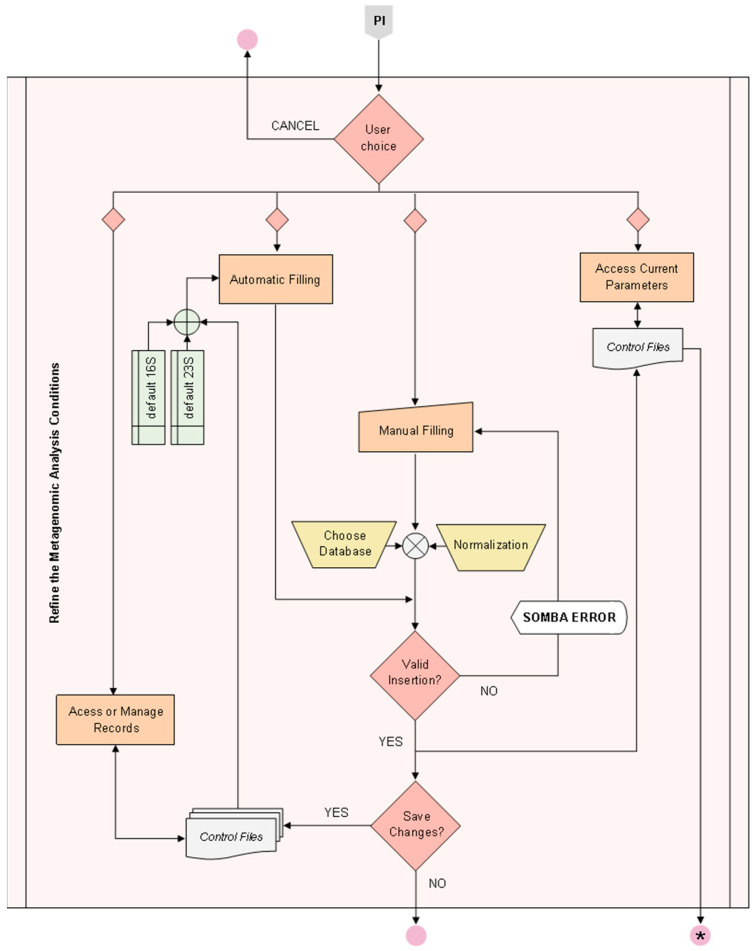
The SOMBA workflow is subdivided into two parts: PII represents only the methodology adopted to optimise metagenomic analysis parameters, and PI represents the remaining methodology.

The overall workflow begins with the careful import and validation of samples, ensuring the integrity and pairing of forward and reverse FASTQ files. Subsequently, the metagenomic analysis proceeds through a series of essential procedures: merging paired reads, trimming primers and adapters, stringent quality and length filtering, dereplicating identical sequences, detecting and removing chimeric amplicons, clustering sequences into OTUs, and finally, assigning taxonomic classifications. The culmination of this processing generates a Metadata Taxonomic Profile (MTP) for each sample, which is then used for downstream biodiversity analyses and exported in an accessible format. The pipeline is designed to be highly configurable, with options for automatic parameter filling, manual adjustment, and the recall of previously used settings, ensuring flexibility for diverse research objectives and data characteristics. Figure 1 shows the SOMBA workflow.

### 3.2. Pre-Processing of Raw Sequencing Data

The initial stages of any metagenomic analysis pipeline are dedicated to the rigorous pre-processing of raw sequencing data. These steps are crucial for removing technical artefacts, enhancing data quality, and preparing the sequences for accurate biological interpretation.

#### 3.2.1. Paired-End Read Merging

The first critical step in processing paired-end sequencing data involves merging the forward and reverse reads. Illumina MiSeq technology generates reads from both ends of a DNA fragment, and when the fragment is shorter than the combined lengths of the forward and reverse reads, the two reads overlap. Merging overlapping paired reads significantly improves the overall quality and length of the resulting amplicon sequence by resolving discrepancies in the overlapping region and effectively reducing the error rate [22]. This process is particularly beneficial, given that sequencing quality typically degrades towards the 3′ end of reads, a region that is often involved in overlaps.

Within SOMBA, demultiplexed paired-end reads are merged using the VSEARCH *--fastq_mergepairs* command. This command is configured with several key parameters to control the merging process:

*--fastq_maxdiffs*: This parameter sets the maximum number of non-matching nucleotides allowed in the overlap region between the forward and reverse reads. A higher value permits more mismatches, potentially recovering more reads but at the risk of incorporating more errors. Conversely, a lower value ensures higher accuracy but may lead to fewer successful merges.
*--fastq_maxdiffpct*: Complementing *--fastq_maxdiffs*, this option defines the maximum percentage of non-matching nucleotides allowed in the overlap region. This percentage-based threshold provides a flexible criterion, especially when dealing with variable overlap lengths, ensuring that the proportion of mismatches remains within acceptable limits relative to the overlap size. Both *--fastq_maxdiffs* and *--fastq_maxdiffpct* strongly influence the success rate and quality of the merged sequences.
*--fastq_minovlen*: This parameter specifies the minimum required overlap length in bases between the forward and reverse reads for a successful merge. An insufficient overlap prevents accurate alignment and merging, making this threshold essential for maintaining the integrity of the fused sequences.

The judicious selection of these parameters is vital. An overly permissive setting might lead to the merging of incorrectly overlapping reads or the retention of low-quality sequence information, potentially generating spurious amplicons. Conversely, overly strict parameters might discard a significant proportion of usable data, reducing overall sequencing depth and potentially impacting the representation of less abundant taxa. The optimisation of these parameters, therefore, involves balancing maximising data retention with ensuring high sequence quality.

#### 3.2.2. Primer and Adapter Trimming

Following read merging, the next crucial pre-processing step is the detection and removal of primer and adapter sequences. These sequences are synthetic oligonucleotides used during PCR amplification and library preparation (primers anneal to the conserved regions of the target gene, and adapters facilitate sequencing on the Illumina platform). They are not part of the biological ribosomal RNA gene itself and must be meticulously removed to prevent their interference with downstream analyses, such as sequence alignment and taxonomic classification. Retention of these artificial sequences can lead to spurious mismatches, incorrect similarity calculations, and ultimately, misclassification of OTUs.

Primers and adapters are removed using the Cutadapt tool, specifically its adapter cut command. This command integrates several important options for precise trimming:

- Quality Trimming (quality-cutoff): Before adapter trimming, low-quality bases at the ends of reads are removed. Illumina reads typically exhibit high quality at the 5′ end but show a decline towards the 3′ end. Therefore, a quality-cutoff parameter (e.g., fixed at a Phred score of 10, as commonly recommended [25]) is applied. This ensures that only high-quality nucleotide stretches contribute to the final sequence, reducing the impact of erroneous base calls on downstream processing.
- Linked Adapters: The pipeline supports the trimming of both forward and reverse nucleotide sequences of linked adapters. These adapters are typically present in the 5′-3′ direction and may contain degenerate bases, as defined by the International Union of Pure and Applied Chemistry (IUPAC) nomenclature. Cutadapt’s ability to interpret IUPAC codes enables flexible, accurate matching of diverse adapter sequences.
- Anchoring: The anchoring option in Cutadapt ensures that trimming occurs only if the adapter is confidently found at a specific end (e.g., the highest-quality 5′ end). If an adapter is anchored, it is marked as mandatory for trimming. If the required adapter is not found, the read will not be trimmed, even if other adapters are present. This stringent approach prevents unintended trimming of biological sequences and ensures trimming occurs only when there is strong evidence of adapter presence.

Effective primer and adapter trimming is paramount. Incomplete removal can lead to artificial inflation of sequence length, interfere with sequence alignments, and obscure true biological variations. Conversely, over-trimming can remove genuine biological sequence data, shortening reads and potentially reducing phylogenetic resolution. Therefore, the Cutadapt parameters are carefully chosen to balance precision and data integrity.

#### 3.2.3. Quality and Length Filtering

Even after read merging and adapter trimming, raw sequencing data often contain suboptimal-quality or inappropriate-length sequences that can confound downstream analyses. Therefore, a comprehensive quality- and length-filtering step is indispensable to ensure that only high-integrity sequences are retained. This process removes reads that are likely to be erroneous or problematic, thereby increasing the reliability of subsequent OTU clustering and taxonomic assignment.

Within SOMBA, reads are filtered using the VSEARCH *--fastq_filter* command, which is configured with four critical parameters:

*--fastq_maxee_rate*: This parameter controls the maximum allowed average expected error rate per sequence. The “expected error” for a read is calculated by summing the error probabilities for all nucleotide positions within that sequence, based on their Phred quality scores (Q scores). Sequences with an average expected error exceeding the specified threshold are discarded. This approach is superior to simply filtering by the minimum base quality score, as it considers cumulative quality across the entire read, providing a more robust measure of overall read quality.
*--fastq_minlen*: This option discards sequences that fall below a specified minimum length threshold. Very short sequences often lack sufficient phylogenetic information for accurate taxonomic assignment or may represent fragmented reads. Defining a minimum length ensures that only reads with adequate information content are carried forward.
*--fastq_maxlen*: Conversely, this parameter discards sequences that exceed a specified maximum length threshold. Sequences that are unusually long may indicate incomplete adapter trimming, chimaeras, or other artefacts. Setting a maximum length helps to maintain consistency in sequence length, which is beneficial for clustering and alignment.
*--fastq_maxns*: This parameter targets sequences containing an excessive number of “N” bases. In the IUPAC nomenclature code, “N” represents any nucleic acid base, indicating an ambiguous base call where the sequencer could not confidently determine the nucleotide. A high proportion of Ns in a read suggests poor quality and unreliability. Sequences with more Ns than the specified threshold are therefore discarded to maintain high data integrity.

Collectively, these filtering parameters work in concert to eliminate low-quality, ambiguous, or abnormally sized sequences. This stringent filtering process is critical for preventing false OTU generation, reducing computational burden in subsequent steps, and ensuring that the final taxonomic profiles are based on biologically meaningful, high-confidence data.

### 3.3. Downstream Metagenomic Analysis

Once the raw sequencing data have been meticulously pre-processed, the pipeline proceeds to the core metagenomic analysis steps, including dereplication, chimaera removal, OTU clustering, and taxonomic assignment. These steps transform the quality-controlled reads into a structured taxonomic profile.

#### 3.3.1. Dereplication of Sequences

The sheer volume of sequences generated by high-throughput platforms often contains a substantial number of strictly identical reads, meaning sequences that have the exact same length and nucleotide string. Retaining all these redundant copies throughout the analysis workflow would unnecessarily increase computational time and memory requirements without adding new biological information. Therefore, a dereplication step is crucial for efficiency and for identifying the unique biological sequences present in the dataset.

Within SOMBA, non-redundant reads are extracted using the VSEARCH *--fastx_uniques* command. This function identifies and groups strictly identical sequences, producing a set of unique sequences, each accompanied by an abundance count indicating how many times that unique sequence appeared in the original dataset. Several parameters are used to refine this process:

*--minuniquesize*: This option is used to discard unique sequences whose post-dereplication abundance value (i.e., the count of identical reads) is smaller than a specified number. Sequences appearing only once or a very few times (singletons or doubletons) might represent rare biological entities, but could also be the result of sequencing errors. Filtering out very low-abundance unique sequences can help reduce noise and focus on more robust signals, although the threshold selection requires careful consideration to avoid discarding genuinely rare taxa.
*--sizeout*: When this option is fixed, VSEARCH adds abundance information to the header of each consensus (unique) sequence. This annotation is critical because it carries quantitative information on the prevalence of each unique sequence, which is essential for downstream analyses such as OTU clustering and diversity calculations.
*--sizein*: By default, the abundance annotations generated by *–sizeout* are considered in subsequent steps of the metagenomic analysis when *–sizein* is fixed. This ensures that the abundance information associated with each unique sequence is consistently utilised throughout the pipeline, particularly during OTU clustering and chimaera detection, where abundance-based heuristics play a significant role.

The dereplication process effectively compresses the dataset, reducing its size without losing critical information about sequence variations and their frequencies. This optimisation significantly improves the pipeline’s computational efficiency, making the analysis of massive sequencing datasets more manageable.

#### 3.3.2. Chimaera Detection and Removal

Chimeric sequences are artificial PCR products that are erroneously generated during amplification when two or more distinct DNA template molecules partially anneal and are extended by the polymerase, resulting in a hybrid sequence composed of segments from different biological origins [28]. The generation of such chimaeras is an inherent, often unavoidable byproduct of NGS amplicon sequencing libraries. If not properly identified and filtered, unverified chimaeras can be mistakenly interpreted as novel species, leading to an overestimation of diversity indices and distorting the true taxonomic profile of a community. Therefore, robust chimaera detection and removal are critical steps in maintaining the precision and accuracy of metagenomic analyses.

Within SOMBA, chimaeras are automatically detected and removed using the VSEARCH *--uchime3_denovo* command. This command implements a de novo chimaera-detection algorithm, meaning it does not rely on a reference database but rather identifies chimaeras by comparing sequences within the input dataset. The algorithm is parameterised with a crucial option:

*--abskew*: The *abskew* (abundance skew) parameter is fundamental for distinguishing between chimeric sequences and their parent sequences in a three-way alignment. The algorithm’s underlying assumption is that chimaeras typically occur later in the PCR amplification process and are thus less abundant than their parent sequences. By analysing the abundance distribution of sequences, *uchime3_denovo* can identify sequences that exhibit chimaera-like characteristics (e.g., being a mosaic of two or more abundant parent sequences). A carefully chosen *abskew* value allows the algorithm to effectively identify and filter these unwanted artefacts.

Removing chimeric sequences is a challenging but indispensable step. Its successful implementation prevents the artificial inflation of species richness and ensures that the subsequent OTU clustering and taxonomic assignments are based on genuine biological sequences. The de novo nature of VSEARCH’s chimaera detection enables the identification of chimaeras even in novel or poorly characterised microbial communities.

#### 3.3.3. Operational Taxonomic Unit Clustering

Following dereplication and chimaera removal, the purified sequences are grouped into OTUs. OTUs serve as pragmatic proxies for species or higher taxonomic ranks, defined purely by a specified percentage of sequence similarity. This clustering approach addresses the challenge of distinguishing between true biological diversity and sequence variation introduced by sequencing errors or intragenomic heterogeneity. A common similarity threshold, such as 97% for prokaryotes or 99% for eukaryotes, is often applied to delineate species-level OTUs [17,18].

Within SOMBA, sequences are clustered into OTUs using a de novo greedy and heuristic centroid-based clustering method implemented by the VSEARCH *--cluster_size* command. This method operates as follows:

- Input Sequence Preparation: The input sequences, which have been previously dereplicated and chimaera-filtered, are typically processed as pre-sorted based on their abundance (as determined during the dereplication step via the *–sizeout* option). Sorting by abundance (e.g., from most to least abundant) can improve the efficiency and accuracy of greedy clustering algorithms, as more abundant sequences are likely to become centroids first.
- Centroid-Based Clustering: The clustering process begins with an empty database of centroid sequences. Each input sequence is then iteratively used as a query:
  – Search for Match: The query sequence is compared against the existing centroids in the database. The search employs a heuristic procedure that prioritises finding the most similar sequences first, optimising the search efficiency.
  – Clustering with Centroid: If the query sequence exhibits a similarity to an existing centroid that is equal to or greater than the predefined *id* (identity) threshold (e.g., *--id* 0.97 for 97% similarity), it is clustered with that centroid. This means it is assigned to the OTU represented by that centroid.
  – New Centroid Creation: If no match meeting the *id* threshold is detected for the query sequence among the existing centroids, the query sequence itself is designated as the centroid of a *new* cluster, and it is added to the database of centroids. This new centroid then represents a novel OTU.

This de novo greedy approach is advantageous because it does not rely on a pre-existing reference database for clustering, making it suitable for exploring novel or less-characterised microbial communities. The choice of the *--id* parameter is critical, as it directly determines the taxonomic resolution of the OTUs. A higher *id* (e.g., 99%) will result in more, smaller OTUs, potentially resolving finer species-level distinctions, while a lower *id* (e.g., 90%) will yield fewer, larger OTUs, grouping sequences at a broader taxonomic level. The clustering process is fundamental for reducing complexity and providing a manageable set of taxonomic units for subsequent classification and diversity analysis.

#### 3.3.4. Taxonomic Assignment

The final step in generating a taxonomic profile involves assigning specific taxonomic labels to each identified OTU. This process links sequence-based OTUs to known biological classifications, enabling the identification of species, genera, families, and higher taxonomic ranks present in the sample. Accurate taxonomic assignment is crucial for interpreting the ecological roles and phylogenetic relationships within a community.

Within SOMBA, the taxonomic classification of each OTU is performed using the VSEARCH *--syntax* command. This command employs a robust method for assigning taxonomy by comparing OTU representative sequences against a curated reference database. The classification process is guided by a specific parameter:

*--syntax_cutoff*: This parameter defines the minimum level of bootstrap support required for a taxonomic assignment to be considered reliable. Bootstrap support is a statistical measure of confidence in a sequence’s phylogenetic placement; a higher cutoff implies a more stringent requirement for classification accuracy. Sequences failing to meet this cutoff for a particular taxonomic rank may be assigned to a higher, more confident rank (e.g., genus instead of species) or remain unclassified at that specific level.

A critical prerequisite for the VSEARCH *--syntax* command within the SOMBA methodology is the precise formatting of the reference database in FASTA format. As previously detailed in Section 2.2, each reference sequence in the database must include comprehensive taxonomic labels within its header, following a strict syntax: >code;tax= p:phylum_name,c:class_name,o:order_name,f:family_name,g:genus_name,s:species_name. This structured format, which includes placeholders for phylum (p), class (c), order (o), family (f), genus (g), and species (s), enables the *syntax* command to parse and assign taxonomy consistently across seven hierarchical levels. Any database used with this pipeline must be pre-formatted to this specification to ensure correct interpretation and reliable classification. This rigorous approach to database formatting and classification parameters ensures that the taxonomic profiles generated are both comprehensive and accurate, forming a solid foundation for ecological interpretation.

### 3.4. Metadata Taxonomic Profile Generation and Data Export

Upon the completion of the core metagenomic analysis steps, including OTU clustering and taxonomic assignment, all pertinent information for each sample is systematically organised into an MTP. This structured data output serves as the comprehensive summary of the taxonomic composition of the community under investigation.

The MTP for each sample encapsulates taxonomic information for all identified OTUs across seven hierarchical levels: phylum, class, order, family, genus, and species. For each OTU at each taxonomic level, the MTP includes its observed abundance (the count of reads assigned to that OTU) and its relative proportion within the sample (the abundance normalised by the total number of reads in the sample). This detailed breakdown provides a granular understanding of community structure, from broad phylogenetic groups to species-level resolution where possible.

A crucial step in preparing the MTP for comparative analyses, particularly when dealing with multiple samples, is data normalisation. Metagenomic sequencing depths can vary significantly across samples due to technical factors, confounding direct comparisons of species richness and abundance. To address this, rarefaction normalisation is performed when deemed appropriate for the study’s objectives. This method involves randomly subsampling reads from each sample until all samples reach a uniform, predefined number of reads. Typically, this predefined number is set to the smallest abundance size among all samples, ensuring that all samples are compared at an equivalent sequencing depth. Rarefaction helps to mitigate the bias introduced by differences in sequencing effort, providing a more robust basis for comparing biodiversity across samples.

Once the metagenomic information is organised and normalised (if applicable), it is exported to a user-friendly Excel file. This design choice aims to facilitate the subsequent analysis and interpretation of results by researchers, making the data accessible without requiring specialised bioinformatics tools. The exported Excel document typically comprises several sheets:

- A primary sheet presenting the full taxonomic profile of all samples at each specified taxonomic level.
- Additional sheets dedicated to various estimated biodiversity indices, calculated at both the genus and species levels. These indices provide quantitative measures of community diversity, richness, and evenness, as further detailed in Section 4.
- Information on the sample ordering method, if any multivariate analysis has been performed.
- Summary statistics, including the total number of target reads (valid reads after stringent quality control) and the total number of identified OTUs for each sample.

The entire process, from the initial import of samples to the final export of the results, is timed by the pipeline. While specific computational times are not presented here (as this paper focuses solely on methodology), the internal tracking of execution time allows for performance assessment and optimisation of the pipeline. This comprehensive MTP generation and export strategy ensures that the complex metagenomic data are translated into an organised, interpretable, and shareable format for downstream ecological and statistical analyses.

To validate the methodology, a sample of wastewater with microalgae from a WWTP in northern Portugal was used, applying the standard parameters described in the following section. The results generated by SOMBA are available in the Supplementary Material.

### 3.5. User Interface for Parameter Optimisation

**Figure 2:**
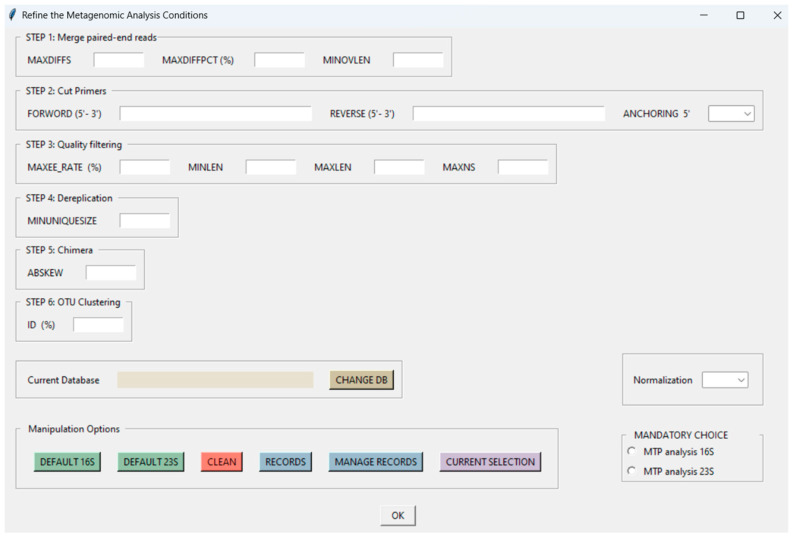
Menu to refine the metagenomic analysis conditions.

Recognising that the optimal parameters for metagenomic analysis can vary significantly depending on the nature of the sequencing data, the specific target gene (16S vs. 23S rRNA), and the research objectives, the SOMBA pipeline incorporates a sophisticated graphical user interface (GUI). This GUI, developed with Python’s Tkinter package (see Figure 2), empowers users with precise control over parameters at each stage of the bioinformatics workflow, thereby directly influencing the quality and specificity of the results.

The GUI, conceptually represented by a menu structure, provides an intuitive, interactive platform for parameter manipulation. This functionality adds substantial value to the program, transforming it from a rigid script into a flexible and adaptable tool. To ensure data integrity and prevent errors from propagating through the pipeline, all input fields in the GUI are validated rigorously. This means that users are prompted to enter valid data types and values within acceptable ranges for each parameter, minimising the likelihood of computational failures or biologically implausible settings.

Parameter filling within the SOMBA GUI can be performed through three distinct mechanisms, catering to different user preferences and workflows:

- Automatic Filling with Default Values: For users seeking a quick start or working with standard datasets, the pipeline provides a set of pre-configured default parameters for each analytical step. These defaults are established based on widely accepted best practices in 16S and 23S rRNA metagenomics, offering a reliable baseline for processing. This option streamlines the setup process, making the pipeline immediately accessible.
- Manual Entry with Storage Capability: Experienced users or those with specific experimental requirements can manually enter customised parameters for each stage of the analysis. This granular control allows for fine-tuning the pipeline to match the unique characteristics of their data or specific research questions (e.g., adjusting the OTU clustering threshold for finer or broader taxonomic resolution). Critically, the GUI offers the functionality to store these customised parameter selections, enabling users to save and recall their preferred settings for future analyses. This feature promotes reproducibility and efficiency when working with similar datasets or repeating analyses.
- Automatic Filling with Previously Processed Records: To further enhance workflow efficiency and consistency, the GUI supports automatic filling of parameters based on settings used in previously processed records. This means that if a user successfully runs the pipeline with a particular set of parameters for one dataset, those exact settings can be automatically applied to a new dataset. This is particularly useful for longitudinal studies or projects involving multiple batches of samples that require identical processing conditions, ensuring methodological consistency across experiments.

An example of the default parameters for each step of the adopted methodology, if presented in a tabular format (e.g., analogous to a detailed Table 1), would clearly delineate the starting configurations for a given study. Similarly, a comprehensive description of additional options available within the GUI (e.g., as detailed in a hypothetical Table 2) would highlight the breadth of customisation available to the user.

The robust design of this user interface significantly lowers the technical barrier for metagenomic data analysis. By providing intuitive control over critical parameters and incorporating error-prevention mechanisms, SOMBA empowers researchers to perform sophisticated bioinformatics analyses with confidence, fostering greater reproducibility and enabling more tailored investigations of microbial and microalgal communities.

## 4. Biodiversity Analysis Methodologies

Beyond characterising the taxonomic composition, a crucial aspect of metagenomic studies involves quantifying and comparing the biodiversity within and between communities. Biodiversity measures provide essential insights into ecological processes, environmental health, and evolutionary patterns. The SOMBA pipeline integrates a comprehensive suite of alpha (α) and beta (β) diversity indices, implemented primarily using the *scikit-bio* package or internal Python code, to offer a thorough ecological perspective.

Biodiversity can be conceptually divided into two main dimensions: alpha (α) diversity and beta (β) diversity. Alpha diversity pertains to the diversity within a single, defined spatial unit or sample, encompassing measures of species richness (the number of distinct species or OTUs) and evenness (the relative abundance distribution among those species). Beta diversity, on the other hand, quantifies the differences in species composition, abundance, or turnover between two or more spatial units or samples [26,27]. While species richness is often perceived as the most straightforward measure of diversity, its direct estimation can be heavily influenced by sampling effort and species detectability. Therefore, a holistic approach requires the use of heterogeneous estimators that integrate both richness and evenness, alongside measures that account for phylogenetic relatedness among species [26]. The choice of the most appropriate biodiversity measures is highly context-dependent, depending on the study’s specific objectives and the inherent characteristics of the sequencing data.

### 4.1. Alpha Diversity Metrics

Alpha diversity metrics quantify the diversity within a single sample or community. They are instrumental in understanding the complexity, structure, and completeness of local microbial or microalgal assemblages.

#### 4.1.1. Species Richness Estimators

Species richness, defined as the total number of different species (or OTUs) observed in a sample, is the most intuitive measure of diversity. However, observed richness is often an underestimate of true richness due to incomplete sampling. Non-parametric estimators attempt to infer the total number of species, including those not observed.

- Margalef Index (*D_Mg_*) [29]: The Margalef index is a simple species richness index that attempts to compensate for the influence of sample size. It divides the number of observed species by the natural logarithm of the total number of individuals, thereby adjusting for sampling effort.
  (1) $$D_{Mg}=\frac{\left(S-1\right)}{\ln N}$$
  where *S* is the total number of observed species (OTUs) in the sample, and *N* is the total number of individuals (reads) in the sample [26]. A higher *D_Mg_* value indicates greater species richness relative to the sample size.

- Menhinick Index (*D_Mn_*) [29]: Similar to the Margalef index, the Menhinick index also aims to normalise species richness by sample size, though it uses the square root of the total number of individuals.
  (2) $$D_{Mn}=\frac{S}{\sqrt{N}}$$
  Both the Margalef and Menhinick indices are sensitive to sampling effort, but provide a basic, size-adjusted measure of richness.

- Chao1 (*S_Chao_*_1_) [30]: The Chao1 estimator, popularised by Colwell and Coddington [31], is a non-parametric method for estimating total species richness based on the numbers of rare species within a sample. It is particularly effective for datasets where many species are represented by only one or two individuals. The primary formula is:
  (3) $$S_{Chao1}=S+\frac{{F_{1}}^{2}}{2F_{2}}$$
  where *F*_1_ is the number of observed species represented by a single individual (singletons), and *F*_2_ is the number of observed species represented by two individuals (doubletons) [26,32,33]. If there are no doubletons (i.e., *F*_2_ = 0), a bias-corrected version is employed:
  (4) $$S_{Chao1}=\;S+\;\frac{F_{1}(F_{1}-1)}{2(F_{2}-1)}$$
  The Chao1 index provides an estimate of the minimum number of species present in a community, including those that were not sampled, based on the assumption that rare species provide the most information about unobserved diversity.

- Jackknife 1 (*S_Jack_*_1_) [34]: The Jackknife 1 estimator is another non-parametric method used to estimate total species richness. It is a first-order jackknife estimator that relies on the number of species that are unique to single samples in a multi-sample dataset.
  (5) $$S_{Jack\;1}=S+Q_{1}\left(\;\frac{m-1}{m}\;\right)$$
  where *Q*_1_ is the number of species found in only one sample (unique species across samples), and *m* is the total number of samples [26,33]. This estimator extrapolates total richness by accounting for species that are so rare they might appear in only a single sampling event.

- Abundance-Based Coverage Estimator (*S_ACE_*) [35]: The Abundance-Based Coverage Estimator (ACE) is a sophisticated non-parametric richness estimator that distinguishes between ‘rare’ and ‘abundant’ species to provide a more accurate estimate of total richness. It recognises that widespread or abundant species offer little insight into unseen diversity, whereas rare species are key indicators. ACE sets a threshold (typically 10 individuals) to classify species.
  (6) $$\begin{array}{l} S_{ACE}=S_{abund}+\frac{S_{rare}}{C_{ACE}}+\frac{F_{1}}{C_{ACE}}{\gamma \;}_{ACE}^{2}\;\;\;\;\;\\ \mathrm{where}\;\;C_{ACE}=1-F_{1}/N_{rare}\;\;\;\; \end{array}$$
  where (i) *S_rare_* is the number of rare species (defined as those with ≤10 individuals); (ii) *S_abund_* is the number of abundant species (defined as those with >10 individuals); (iii) *N_rare_* is the total number of individuals (reads) in rare species; (iv) *F_i_* is the number of species with i*i* individuals (*F*_1_ is the number of singletons); (v) *C_ACE_* is the estimated sample coverage for rare species; and (vi) *γ_ACE_*^2^ is an estimator for the coefficient of variation in the *F_i_*’s among rare species, defined as:
  (7) $${\gamma \;}_{ACE}^{2}=max\left\{\frac{S_{rare}}{C_{ACE}}\frac{\sum_{i=1}^{10}i(i-1)F_{i}}{N_{rare}(N_{rare}-1)}-1\right\}$$
  The ACE index provides a robust estimate by giving preponderant weight to the contribution of uncommon species, offering valuable insight into the wealth of a community [26,36].

#### 4.1.2. Heterogeneity and Evenness Indices

While richness measures quantify the number of species, heterogeneity and evenness indices provide insights into how individuals are distributed among those species. A community with high evenness has species of roughly equal abundance, whereas a community with low evenness is dominated by a few species.

- Shannon Index (*H*′) [29]: The Shannon index (also known as the Shannon–Weaver or Shannon–Wiener index) is a widely used measure of diversity that quantifies both species richness and evenness. It is based on information theory, assuming that individuals are randomly sampled from an infinitely large population and that all species are represented in the sample. A higher value of *H*′ indicates greater diversity.
  (8) $$H^{\prime }=-\sum p_{i}\;\log_{2}p_{i}$$
  where *p_i_* is the proportion of individuals belonging to species *i*, estimated as *ni*/*N* (where *n_i_* is the abundance of species *i*, and *N* is the total number of individuals in the sample) [23,27]. The base of the logarithm can vary (e.g., natural logarithms or base-10 logarithms), but base 2 is commonly used in bioinformatics.

- Pielou’s Evenness (*J*′) [37]: Pielou’s evenness index normalises the Shannon index to range between 0 and 1, providing a direct measure of how evenly distributed species abundances are. It is calculated as the ratio of the observed Shannon diversity to the maximum possible diversity, which occurs when all species have equal abundances.
  (9) $$J^{\prime }=\frac{H^{\prime }}{H_{max}}=\frac{H^{\prime }}{\ln S}\;$$
  where *H_max_* represents the maximum diversity if all *S* species were equally abundant. A value of *J*′ close to 1 indicates high evenness, while values closer to 0 suggest dominance by a few species [26,38].

- Heip’s Evenness (*E_Heip_*) [39]: Heip’s evenness index is another measure designed to be less dependent on species richness, particularly for larger samples. It aims to yield low values when evenness is distinctly poor, irrespective of the number of species.
  (10) $$E_{Heip}=\frac{\left(e^{H^{\prime }}-1\right)}{\left(S-1\right)}$$
  While it may not be entirely independent of sample size for very small communities (e.g., fewer than 10 species), it generally satisfies the criterion of reflecting true evenness [26,40].

- Brillouin Index (*HB*) [41]: The Brillouin index is an alternative measure of heterogeneity, particularly suited for situations where the randomness of sampling cannot be guaranteed, or when the entire community (a “known collection”) has been exhaustively counted. Unlike the Shannon index, which estimates diversity for an indefinitely large population from a sample, the Brillouin index describes the diversity of the specific, finite collection observed.
  (11) $$HB=\frac{\ln N!-\sum \ln n_{i}!}{N}$$
  The Brillouin index will always yield a lower value than the Shannon index for the same data because it quantifies the diversity of a precisely known collection with no uncertainty [26]. Despite its mathematical superiority for non-random collections, it is less widely used due to higher computational demands and lesser familiarity compared to the Shannon index.

#### 4.1.3. Dominance Measures

Dominance measures focus on the inverse of evenness, quantifying the extent to which a few species dominate the community in terms of abundance.

- Simpson Index (*D*) and Simpson’s Evenness (*E*_1/*D*_) [42]: The Simpson index is one of the oldest and most widely recognised measures of dominance. It quantifies the probability that two individuals randomly selected from a sample are members of the same species.

A higher value of *D* indicates greater dominance (less diversity). Therefore, it is often expressed as its inverse, 1/*D*, or 1 − *D* (Gini-Simpson index) [43], where higher values indicate greater diversity. A related measure, Simpson’s evenness, normalises the inverse Simpson index by species richness:
  (12) $$HB=\;\frac{\ln N!-\;\sum \ln n_{i}!}{N}$$
  (13) $$E_{1/D}=\frac{\left(1/D\right)}{S}$$
  This metric provides a sense of evenness adjusted by the overall number of species [26].

- Berger-Parker Index (*d*) [41]: The Berger-Parker index is a simple and intuitive measure of dominance that directly expresses the proportional abundance of the single most abundant species in a sample.
  (14) $$d=\frac{N_{max}}{N}$$
  where *N_max_* is the number of individuals in the most abundant species. A higher value of *d* indicates greater dominance by a single species. This index is generally independent of species richness for large assemblages (over 100 species) but tends to decrease with increasing richness in smaller assemblages [26,44].

#### 4.1.4. Phylogenetic Diversity

Traditional diversity measures often treat all species as equally distinct. However, if two communities have the same number of species and similar abundance patterns but differ in the phylogenetic relatedness of their constituent species, the one with more phylogenetically distinct species might be considered more diverse [45]. Phylogenetic diversity (PD) explicitly incorporates evolutionary information into diversity assessments.

- Faith’s PD [46]: Its measure quantifies taxonomic diversity by summing the cumulative branch lengths of a phylogenetic tree that connects all species within a given sample [47,48]. This approach accounts for the evolutionary history and genetic divergence among species, providing a more comprehensive understanding of diversity than species counts alone. Communities with older or more distantly related lineages will exhibit higher PD, even when species richness is similar to that of a community with closely related species. The calculation of PD requires a phylogenetic tree constructed from the representative sequences of the OTUs.

#### 4.1.5. Sampling Completeness Assessment

Assessing whether the sampling effort has adequately captured the true diversity of a community is vital for the reliability of any biodiversity analysis.

- Good Coverage (GC) Index: The GC index provides a direct measure of sampling completeness, indicating the extent to which the observed sequencing reads represent the actual population of species in the sample.
  (15) $$GC=1-\frac{F_{1}}{N}$$
  The value of GC ranges from 0 to 1 (or 0% to 100%). A GC value close to 1 (100%) suggests that the sequencing effort has effectively sampled the majority of species, implying that additional sequencing is unlikely to reveal many new species. Conversely, a lower GC value indicates that a substantial proportion of the community’s diversity remains unsampled [49]. This index provides a critical quality-control metric for evaluating the adequacy of sequencing depth.

### 4.2. Beta Diversity Metrics

Beta diversity measures quantify the differences in species composition and/or abundance between two or more communities or samples. They reveal how community structure changes across spatial or temporal gradients and are fundamental for understanding ecological patterns such as complementarity, turnover, and biotic distinctness [50].

#### 4.2.1. Community Similarity and Dissimilarity Coefficients

These measures compare communities based on the presence or absence of species.

- Jaccard Index (*C_J_*) [51]: The Jaccard index is a classic and widely used similarity coefficient that quantifies the overlap in species composition between two samples. It is calculated as the ratio of the number of species common to both samples to the total number of species found in either sample.
  (16) $$C_{J}=\frac{a}{a+b+c}\;$$
  where *a* is the number of species present in both samples (m_1_ and m_2_), *b* is the number of species present only in sample m_1_, and *c* is the number of species present only in sample m_2_ [26,52]. The Jaccard index ranges from 0 (no shared species) to 1 (identical species composition). A limitation of presence/absence-based measures like Jaccard is that they do not account for species’ relative abundances; a dominant species counts as much as a rare singleton.

#### 4.2.2. Quantitative Abundance-Based Measures

To address the limitations of presence/absence metrics, quantitative measures incorporate species abundance information, providing a more nuanced comparison between communities.

- Bray–Curtis Index (*C_N_*) [53]: The Bray–Curtis index is a popular dissimilarity measure that accounts for species abundance. It is calculated as the sum of the absolute differences in species abundances between two communities, normalised by the total abundance in both communities. The pipeline computes its complement, the similarity index.
  (17) $$C_{N}=\frac{2jN}{N_{{\;m}_{1}}+N_{{\;m}_{2}}}$$
  where 2*jN* is the sum of the lower of the two abundances for species found in both samples m_1_ and m_2_, and *N_m_*_1_ and *N_m_*_2_ are the total number of individuals (reads) in samples m_1_ and m_2_, respectively [54]. This index ranges from 0 (no shared species or vastly different abundances) to 1 (identical species composition and abundances).

- Pearson’s Correlation Coefficient: Pearson’s correlation coefficient is a statistical measure that quantifies the linear relationship between two variables. In the context of beta diversity, it can be applied to assess the strength and direction of the relationship between species abundance profiles in two different samples.
  (18) $$r=\frac{\sum \left(n_{i,{\;m}_{1}}-\bar{n_{i,{\;m}_{1}}})(n_{i,{\;m}_{2}}-\bar{n_{i,{\;m}_{2}}}\right)}{\sqrt{\sum {\left(n_{i,{\;m}_{1}}-\bar{n_{i,{\;m}_{1}}}\right)}^{2}\;\sum {\left(n_{i,{\;m}_{2}}-\bar{n_{i,{\;m}_{2}}}\right)}^{2}}}$$
  where *n_i_*_,*m*1_ and *n_i_*_,*m*2_ are the abundances of species *i* in samples m_1_ and m_2_, respectively [55]. The coefficient *r* ranges from −1 to 1. A value of *r* > 0 indicates a positive correlation (species abundances co-vary in the same direction), *r* < 0 indicates a negative correlation (one increases as the other decreases), and *r* ≈ 0 indicates a weak or no linear relationship. In metagenomics, it can indicate similar community dynamics or responses between two samples.

#### 4.2.3. Phylogenetic Distance Metrics

Phylogenetic distance measures leverage the evolutionary relationships among species, providing a more powerful and biologically meaningful comparison of communities than methods based solely on species lists or abundances. UniFrac distances are particularly prominent in this category [56].

- UniFrac Distances: UniFrac distances quantify the phylogenetic distance between communities by considering the amount of unique evolutionary history represented by each community in a phylogenetic tree. It requires a rooted phylogenetic tree of all observed OTUs. Let *n* be the number of branches in the tree, *bi* be the length of branch *i*, and *p_i_*_,*A*_ and *p_i_*_,*B*_ be the proportions of taxa descended from branch i*i* in communities A and B, respectively.
  – Unweighted UniFrac Distance (*d^U^*): The unweighted UniFrac distance is a qualitative measure that focuses solely on the presence or absence of species. It quantifies the fraction of the total branch length in the phylogenetic tree that leads to descendants found in one community or the other, but not both. It is particularly informative when communities differ primarily in which taxa they support.
    (19) $$d^{U}=\sum_{i=1}^{n}\frac{b_{i}|I\left(p_{i,A}>0\right)-I(p_{i,B}>0)|}{\sum_{i=1}^{n}b_{i}}$$
    where *I*(⋅) is an indicator function that returns 1 if the condition is true (i.e., species descended from branch *i* are present in the community) and 0 otherwise [36,38].
  – Weighted UniFrac Distance (*d^W^*): The weighted UniFrac distance is a quantitative measure that incorporates species abundance information. It weights each branch length by the difference in the relative abundances of its descendants between the two communities. This makes it ideal for revealing variations caused by changes in the relative abundance of taxa.
    (20) $$d^{W}=\frac{\sum_{i=1}^{n}b_{i}\;|p_{i,A}-p_{i,B}|}{\sum_{i=1}^{n}b_{i}\;(p_{i,A}+p_{i,B})}$$
    The numerator uses the absolute proportion difference, meaning *d^W^* values are predominantly influenced by branches with large proportions. This can make it less sensitive to changes in abundance on branches with small proportions [57,58].
  – Normalised Weighted UniFrac Distance (d^W,norm^). To offset the dominance of high-abundance branches in the standard weighted UniFrac, a normalised version is often used. This metric employs relative differences in proportions, giving equal focus to each branch irrespective of its overall abundance, as these differences are less affected by the absolute values of *p_i, A_*, and *p_i, B_*.
    (21) $$d^{W,\;norm}=\frac{\sum_{i=1}^{n}b_{i}\;\left|\frac{\;p_{i,A}-p_{i,B}\;}{p_{i,A}+p_{i,B}}\right|}{\sum_{i=1}^{n}b_{i}}$$
    The denominator acts as a normalising factor, ensuring that *d^W^*^,*norm*^ ∈ [0, 1] [57,58]. This normalised version offers a balanced perspective on phylogenetic community dissimilarities, highlighting both major and subtle shifts in taxonomic composition and abundance.

### 4.3. Multivariate Ordination for Community Visualisation

After calculating various beta diversity measures, particularly distance metrics between samples, visualising these dissimilarities becomes essential for identifying patterns, grouping similar communities, and understanding ecological relationships. The high dimensionality of metagenomic community profiles poses a challenge for direct visualisation.

- Principal Coordinate Analysis: Principal Coordinate Analysis (PCoA), also known as classical multidimensional scaling, is a multivariate ordination technique used to represent a set of objects (e.g., samples) in a reduced-dimensional Euclidean space. The primary goal of PCoA is to preserve the original distance relationships between these objects as much as possible in a lower-dimensional projection, typically 2D or 3D. The method operates by taking a square matrix of dissimilarities (or similarities) between objects (such as a UniFrac distance matrix) as input. It then generates a new set of orthogonal variables, termed Principal Coordinates (PCs), which represent new combinations of the original variables. Each PC successively explains the maximum possible variance in the distance relationships. The first PC accounts for the largest proportion of variance, the second for the next largest, and so on. For the relationships between objects to be accurately represented in Euclidean space, the input distance matrix must be metric, meaning it must satisfy the triangle inequality. UniFrac distance measurements, both unweighted and weighted variations, fulfil this requirement, making them suitable inputs for PCoA [59]. Within SOMBA, three-dimensional PCoA coordinates are determined for both unweighted and weighted UniFrac distances, calculated at both the species and genus levels. These coordinates can then be used to generate 2D or 3D scatter plots, allowing researchers to visually identify clusters of samples with similar community structures and interpret the ecological factors driving these patterns. PCoA is a powerful tool for exploring beta diversity patterns and summarising complex community dissimilarities in an interpretable graphical format.

## 5. Conclusions

The growing reliance on high-throughput sequencing in environmental microbiology and biotechnology has intensified the need for accessible, reliable bioinformatics tools. The SOMBA pipeline presented here offers a comprehensive, transparent framework for processing 16S and 23S rRNA paired-end metabarcoding data, guiding users from raw sequence preprocessing to detailed taxonomic and biodiversity outputs. Built on robust open-source tools such as VSEARCH, Cutadapt, and scikit-bio, SOMBA delivers efficient sequence handling, rigorous quality control, and extensive ecological analyses while maintaining full reproducibility and cost-effectiveness.

A major strength of the system is its user-focused design. Through a Tkinter-based graphical interface, researchers can intuitively adjust parameters, apply default or recalled settings, and ensure data integrity through strict input validation. The pipeline effectively addresses key challenges in metabarcoding workflows, including read merging, primer removal, quality filtering, dereplication, chimaera detection, OTU clustering, and taxonomic assignment using curated databases such as EZBioCloud and µgreen. Automated generation of MTPs and Excel-ready outputs further streamlines downstream interpretation.

Given the inherent variability of bioinformatics pipelines, SOMBA’s explicit documentation and configurable methodology promote transparency and reproducibility. Its flexible, modular structure positions it as a valuable tool for microbial and microalgal community analysis, with future developments aimed at expanding marker compatibility and integrating advanced analytical features.

The design of this convenience tool enables an innovative, simultaneous analysis of both markers from the same samples. Future studies in this direction will be interesting and motivate our team.

We encourage the scientific community to make use of our tool and to test its applicability in their respective research contexts. We remain available to provide support in this regard where needed. We also welcome any feedback or suggestions, which are valuable to further improve the tool and ensure its continued relevance to emerging scientific and technological advances.

## Figures and Tables

**Table 1:** Default parameters for each step of the methodology adopted, where those indicated with 1 and 2 are referenced in ref. [22] and ref. [25], respectively.

Metagenomic Analysis	Parameters	Default 16S	Default 23S
Step 1: Merge paired-end reads	--maxdiffs ^1^	199	18
	--maxdiffpct ^1^	25	10
	--minovlen ^1^	5	100
Step 2: Cut Primers	Linked Adapters ^2^	Forward primer Bakt_341F [23] 5′-CCTACGGGNGGCWGCAG-3′	Forward primer p23SrV_f1 [13] 5′-GGACAGAAAGACCCTATGAA
		Reverse primer Bakt_805R [23] 5′-GACTACHVGGGTATCTAATCC-3	Reverse primer p23SrV_r1 [13] 5′-TCAGCCTGTTATCCCTAGAG-3
	Anchoring 5′ ^2^	True	True
Step 3: Quality Filtering	--maxee_rate ^1^	1	5
	--minlen ^1^	100	100
	--maxlen ^1^	2000	2000
	--maxns ^1^	0	0
Step 4: Dereplication	--minuniquesize ^1^	1	1
Step 5: Chimera detection and removal	--abskew ^1^	16	16
Step 6: Clustering	--ID ^1^	97	97
Current Database		EZBioCloud	µgreen
Normalization		True	True

**Table 2:** Additional features offered by the menu to refine the metagenomic analysis conditions.

Additional Features	
Default 16S	Fills all fields with the default values from the MTP analysis 16S automatically.
Default 23S	Fills all fields with the default values from the MTP analysis 23S automatically.
Clean	Clear all fields automatically.
Records	Allows access to saved records and automatically fills in fields.
Manage Records	To manage records by deleting those that are not necessary, those that contain errors or those that were saved by mistake. For safety reasons, when this option is selected, the menu is deactivated. To re-optimise parameters, close and reopen the menu.
Current Selection	Allows access to the parameters currently in use.
Mandatory Choice	MTP analysis 16S or 23S

## Data Availability

The raw data supporting the conclusions of this article will be made available by the authors on request.

## Supplementary Materials

The following supporting information can be downloaded at: https://www.mdpi.com/article/10.3390/biotech15020042/s1.
