# Supplementary material for: Bioinformatics Strategy for 16s and 23s rRNA Metabarcoding Data
Source: BioTech (Basel). 2026 Jun 8;15(2):42. doi: 10.3390/biotech15020042 (PMC13296876; doi:10.3390/biotech15020042)
Supplement: Supplementary file 1 [file biotech-15-00042-s001.zip › biotech-4293793-supplementary/SOMBA.zip!/SOMBA_UserGuide.pdf]

SOMBA

Software For Metabarcoding Analysis

SOMBA was developed by Rita Domingues < domingues.anarita@gmail.com >

September 2023 [version 1.0]

SOMBA

Software for **M**eta**b**arcoding **A**nalysis (SOMBA) was developed to bridge the gap between metagenomics data and the end user by being independent, user-friendly, running locally where data does not need to be transferred to external servers, and the algorithm does not limit the number of samples that can be executed in a single batch. It is currently designed for processing paired-end sequencing data from the 16S (V3-V4 domain) and 23S rRNA (V5 domain) genes obtained from the Illumina MiSeq^{TM} technology. The pipeline for metagenomics analysis was developed in Python 3.11 using VSEARCH 2.22.1 [1] and Cutadapt 4.4 [2]. For detailed scientific information about the methodology on which SOMBA was based, an article is being written. However, this manual offers a basic guide to using SOMBA, where the following topics will be covered: (1) Program installation and Environment creation, (2) SOMBA workflow overview, (3) Refine the metagenomic analysis conditions and (4) System error codes.

1. Program installation and Environment creation

SOMBA is an executable version, meaning it can run on any operating system free from the dependencies on which the program was developed. The sole requirement for proper execution is that the SOMBA environment be created using the installation procedure. As this is a delicate procedure, installation is carried out by a qualified professional.

As shown in Figure 1, this environment has all the directories that SOMBA needs to run, namely external dependencies and control files. **Under no circumstances should the environment be modified.** Furthermore, **control files must not be opened** in order to avoid adding or removing characters, modifying the information contained in them, and consequently compromising the correct execution of SOMBA. The **SOMBA.exe program must be run directly from the desktop** (in the same directory as the SOMBA folder). The readme file provides additional information about that.

Samples in fastq format must be entered manually by the user in the respective *fastq_input_16S* or *fastq_input_23S* directory. According to the Illumina sequencer, the software receives the names of the files in the format *sample-name_1.fastq* (forward) and *sample-name_2.fastq* (reverse). For the sake of integrity, no space “ ” should be utilized. The results automatically generated by SOMBA are accessed by the user in the *output_16S* or *output_23S* folders accordingly. To avoid overlapping results, the generated files are numbered, increasing the count after successive analyses. By default, in the *Database* directory, the program has the EZBioCloud [3] and µgreen [4] databases for analyzing microbial and microalgae communities, respectively. Other databases can be placed in the corresponding folder as long as they comply with SOMBA requirements, as mentioned in Section 3.

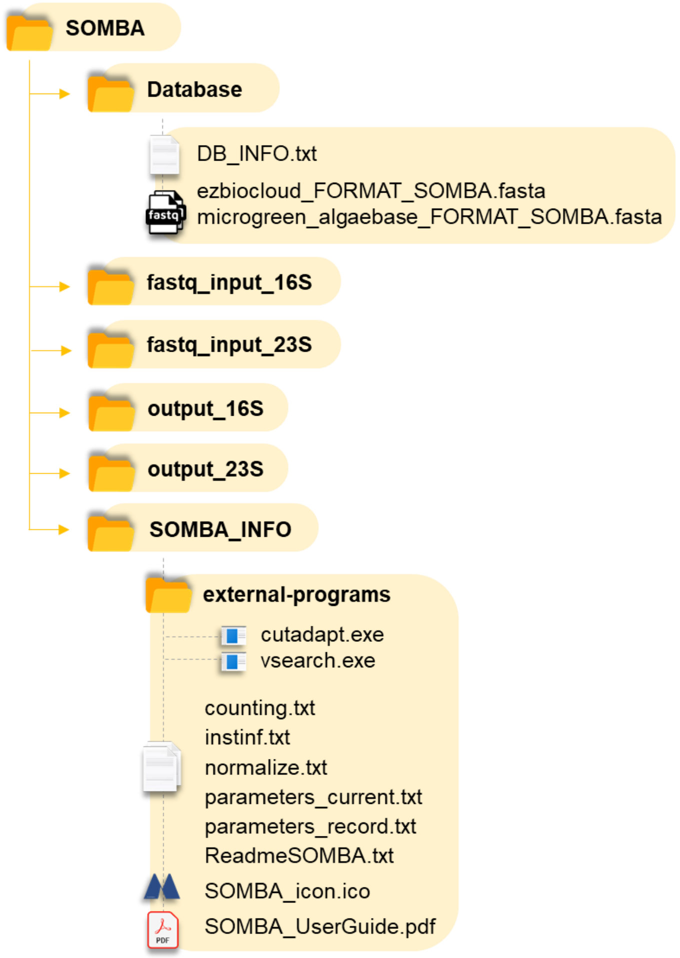

Figure 1. SOMBA environment.

2. SOMBA workflow overview

Figure 2 illustrates the SOMBA methodology to help understand how it works. The Illumina readings (demultiplexed paired-end reads in fastq format) imported from the respective directory into SOMBA are pre-processed (merging, primer cutting, filtering and quality control), keeping only high-quality sequences. The open-reference approach was used to cluster the pre-processed sequencing reads into OTUs, using the database selected according to the type of analysis. SOMBA includes an interactive menu to optimize metagenomic analysis parameters, which is detailed in Section 3. The taxonomic profile characterization procedure is applied to each sample individually. Data processing creates a final Metadata Taxonomy Profile (MTP) that stores, for each taxonomy level, the respective taxonomy, count and abundance information associated with each sample. Alpha and beta diversity measures are estimated for this MTP set. A multivariate analysis is performed using principal coordinate analysis through the distance matrix data resulting from the beta diversity analysis to give the major axes of principal

components (PCs). Up to the first three PCs are supplied, allowing you to draw scatterplots in up to three dimensions. At the end, the results are exported to the respective output directory in a single Excel file. SOMBA is structured with process controls that ensure the correct execution: information, warnings, and error messages are included.

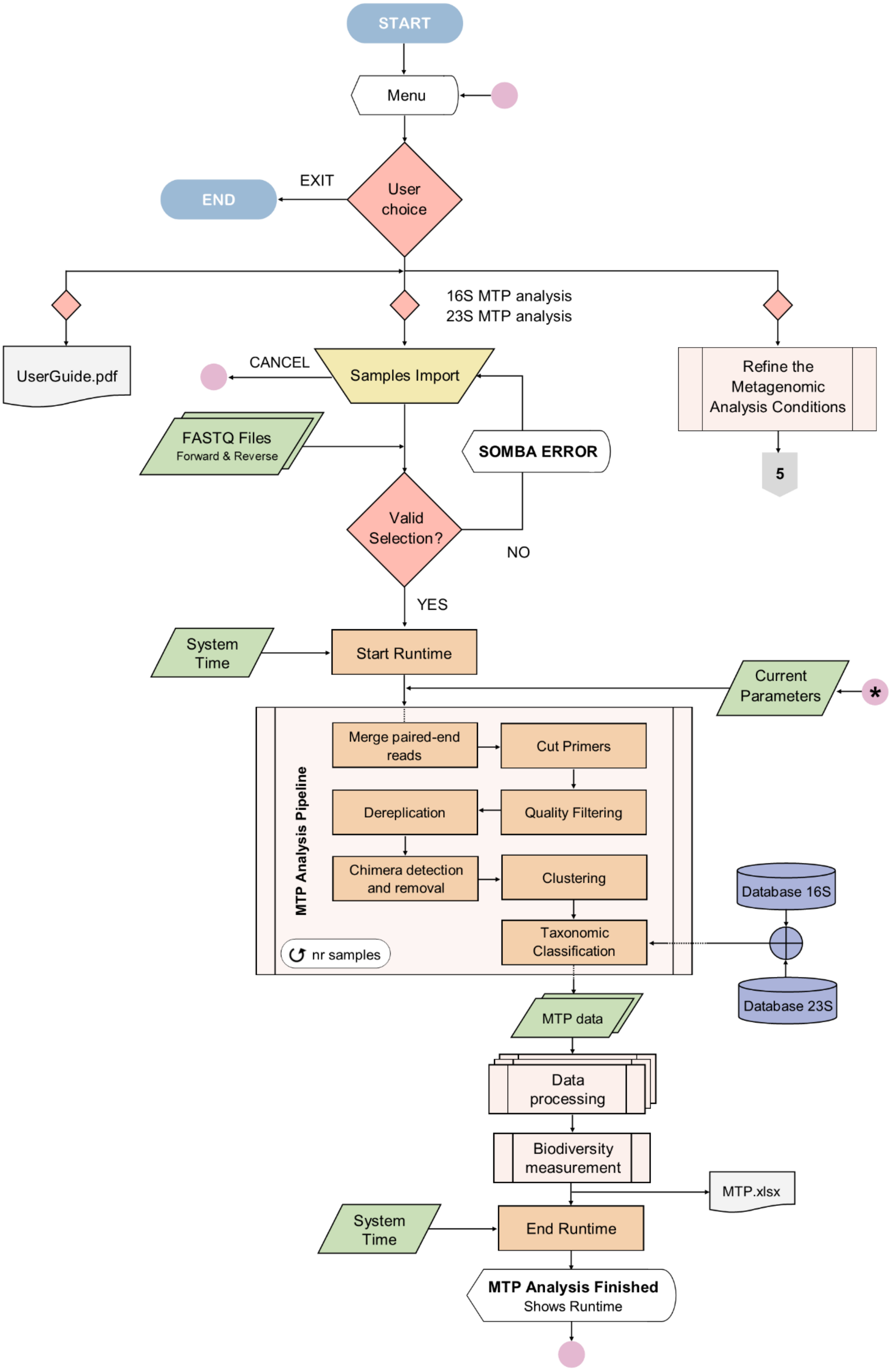
START
Menu
EXIT
END
User choice
16S MTP analysis
23S MTP analysis
UserGuide.pdf
CANCEL
Samples Import
Refine the Metagenomic Analysis Conditions
FASTQ Files
Forward & Reverse
SOMBA ERROR
5
Valid Selection?
NO
YES
System Time
Start Runtime
Current Parameters
*
MTP Analysis Pipeline
Merge paired-end reads
Cut Primers
Dereplication
Quality Filtering
Chimera detection and removal
Clustering
Database 16S
Taxonomic Classification
nr samples
Database 23S
MTP data
Data processing
Biodiversity measurement
MTP.xlsx
System Time
End Runtime
MTP Analysis Finished
Shows Runtime

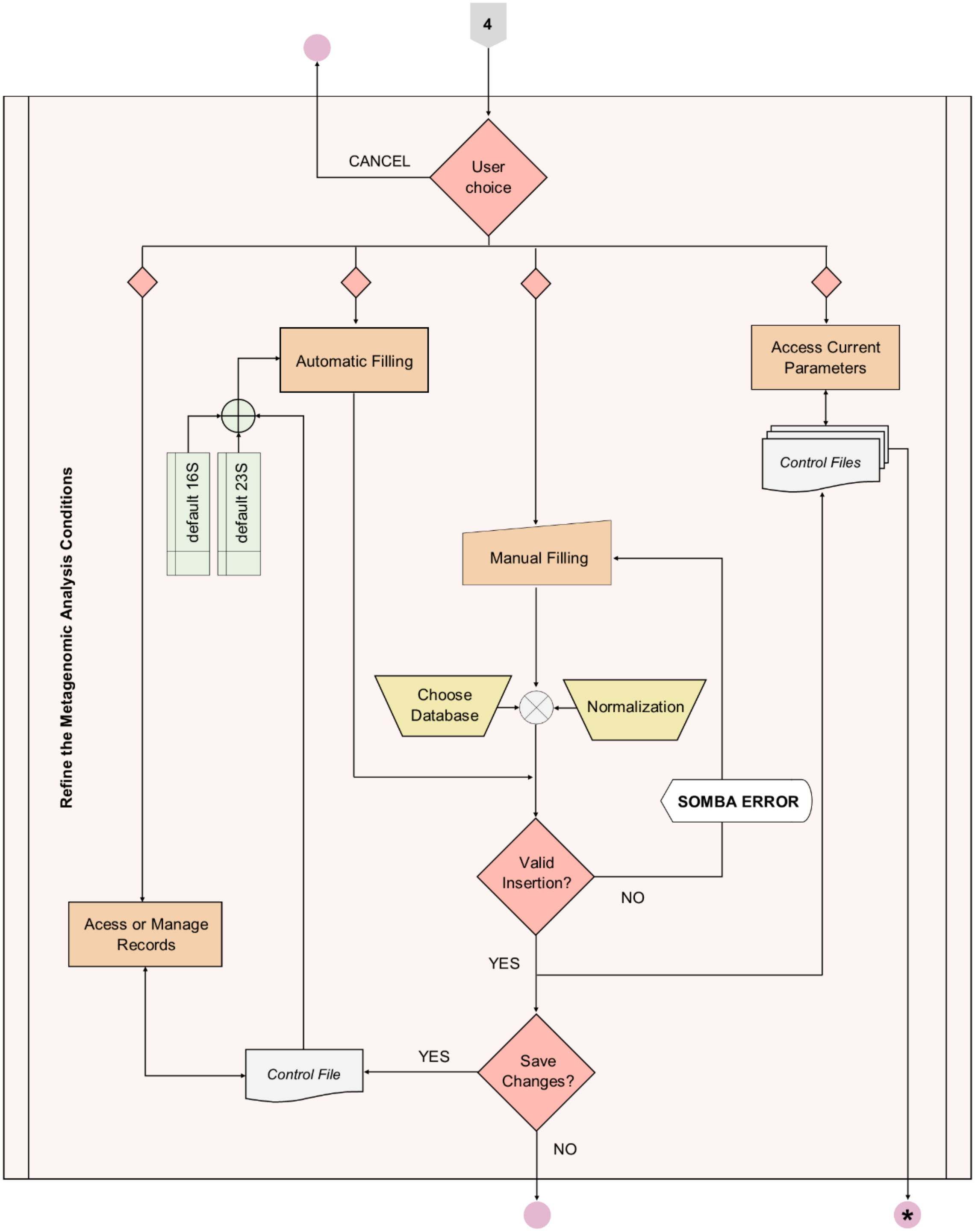

Figure 2. SOMBA workflow.

3. Refine the Metagenomic Analysis Conditions

SOMBA has an interactive menu to optimize metagenomic conditions by manipulating parameters at each step, allowing users to adjust the pipeline to the nature of the sequencing data (Figure 3). This functionality adds value to the program, as it is directly related to the quality of the outcomes. To create a refinement, all fields are mandatory and are delineated by barriers that prevent errors from entering the pipeline. Furthermore, the menu offers several additional options that facilitate this refinement process, including saving changes to a control file. Table 1 brings together the information needed to use this menu, where the reference to each metagenomic parameter was obtained from the VSEARCH and CUTADAPT manual content.

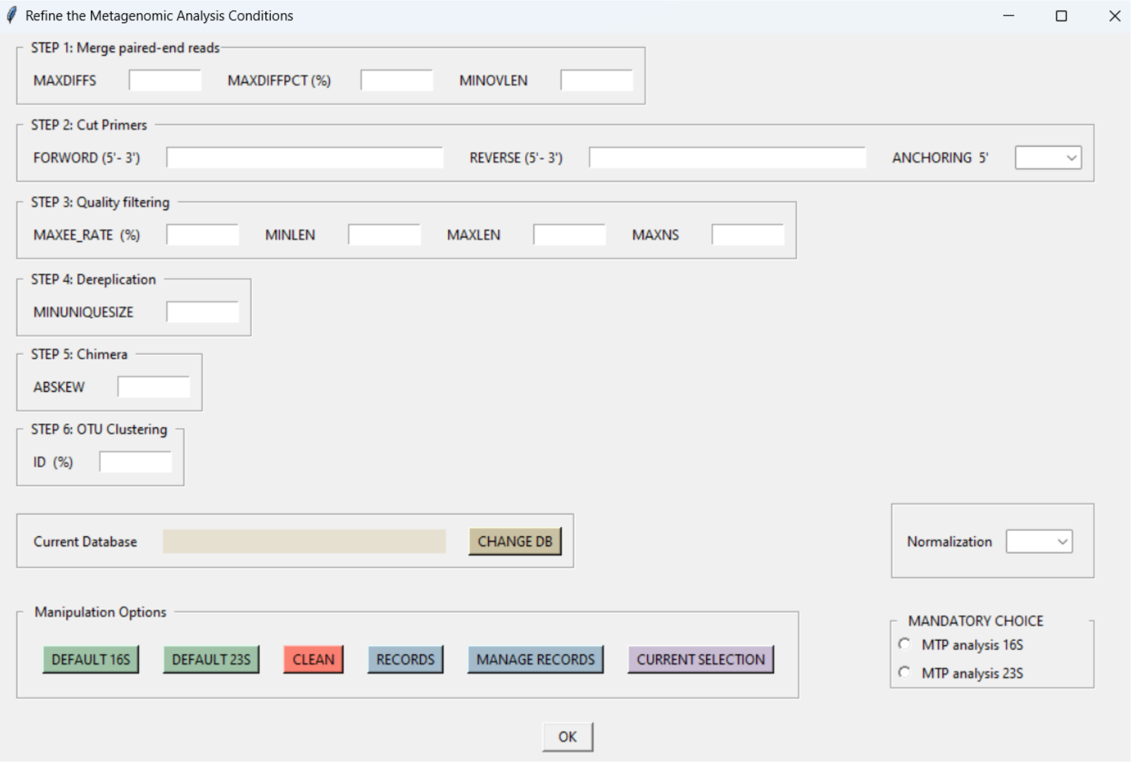

Figure 3. Menu to refine the metagenomic analysis conditions.

Table 1. Menu features.

**Step 1: Merge paired-end reads**		
**maxdiffs**	Specify the maximum number of non-matching nucleotides allowed in the overlap region. That option has a strong influence on the merging success rate.	Positive integer ≥ 1
**maxdiffpct**	Specify the maximum percentage of non-matching nucleotides allowed in the overlap region.	Positive integer in range [1:100]%

continues on the next page

**minovlen**	Specify the minimum overlap between the merged reads. Must be at least 5.	Positive integer ≥ 5
**Step 2: Cut Primers**		
**Linked Adapters**	Forward and reverse nucleotide sequences combined, both in the 5'-3' direction.	IUPAC nomenclature code
**Anchoring 5'**	If an adapter is anchored, it will also become marked as being required. If a required adapter cannot be found, the read will not be trimmed at all, even if the other adapter occurs. The parameter only allows to anchor the highest quality end 5'.	True or False
**Step 3: Quality Filtering**		
**maxee_rate**	Discard sequences with an average expected error greater than the specified number. For a given sequence, the average expected error is the sum of error probabilities for all the positions in the sequence, divided by the length of the sequence.	Positive integer in range [1:100]%
**minlen**	Discard sequences with less than the specified number of bases.	Positive integer ≥ 1
**maxlen**	Discard sequences with more than the specified number of bases.	Positive integer ≥ 1
**maxns**	Discard sequences with more than the specified number of N’s.	Positive integer ≥ 0
**Step 4: Dereplication**		
**minuniquesize**	Discard sequences with a post-dereplication abundance value smaller than specified.	Positive integer ≥ 1
**Step 5: Chimera detection and removal**		
**abskew**	The **ab**undance **skew** is used to distinguish in a threeway alignment which sequence is the chimera and which are the parents. The assumption is that chimeras appear later in the PCR amplification process and are therefore less abundant than their parents.	Positive integer ≥ 1
**Step 6: Clustering**		
**ID**	ID represents the minimum similarity threshold between nucleotide sequences.	Positive integer in range [1:100]%

continues on the next page

Current Database

Shows the database being used and allows loading from the SOMBA environment directory any file in fasta format, with the taxonomic information in the required formatting: ">code;tax= p:phylum_name,c:class_name,o:order_name,f:family_name,g:genus_name,s:species_name". As demonstrated in this format, the taxon name must be spaced using "_" (other characters such as "-" or " " [= space] are not accepted by the software).

Normalization

Samples are normalized by the rarefaction method. This option should be enabled to make diversity measures independent of sample size.	True or False

Manipulation Options

**Default 16S**	Fills all fields with the default values from the MTP analysis 16S automatically.
**Default 23S**	Fills all fields with the default values from the MTP analysis 23S automatically.
**Clean**	Clear all fields automatically.
**Records**	Allows to access saved records and automatically fill in fields.
**Manage Records**	To manage records by deleting those that are not necessary, those that contain errors or those that were saved by mistake. For safety reasons, when this option is selected, the menu is deactivated. To re-optimize parameters, close and reopen the menu.
**Current Selection**	Allows access to the parameters that are currently being used.

Mandatory Choice

MTP analysis 16S or 23S

OK

By clicking this button, the values are automatically validated for the current analysis.

Attention:

- If the wrong parameters selection is validated, do not save the changes and return to this menu for the correct selection.
- If a selection of parameters already saved in the file is run, after "OK" validation, do not save it again.
- If the same parameter selection is saved, access record management and delete repeated records.

4. System error codes

As mentioned, SOMBA is structured with process controls that guarantee correct execution. Information, warnings, and error messages are included. Among them, listed below, are the system error codes that are sent by the program in compromising situations.

Error Code 000
Access denied. The system validation file instinf.txt was not found. Request technical assistance.

Error Code 001
Access denied. The license has expired. Request technical assistance.

Error Code 002
Mandatory external program VSEARCH not found. Request technical assistance.

Error Code 003
Mandatory external program CUTADAPT not found. Request technical assistance.

Error Code 100
File not found. Request technical assistance

Error Code 101
Control file parameters_current.txt not found. Request technical assistance.

Error Code 102
Control file parameters_records.txt not found. Request technical assistance.

Error Code 103
Control file counting.txt not found. Request technical assistance.

Error Code 104
Control file DB_INFO.txt not found. Request technical assistance.

Error Code 105
Control file normalize.txt not found. Request technical assistance.

Error Code 200
ValueError.

Error Code 250
Wrong Selection.

References

[1] T. Rognes, T. Flouri, B. Nichols, C. Quince and F. Mahé, "VSEARCH: a versatile open source tool for metagenomics," *PeerJ,* 16 Oct 2016.

[2] M. Martin, "Cutadapt Removes Adapter Sequences From High-Throughput Sequencing Reads," *EMBnet.journal,* vol. 17, no. 1.

[3] S.-H. Yoon, S.-M. Ha, S. Kwon, J. Lim, Y. Kim, H. Seo and J. Chun, "Introducing EzBioCloud: a taxonomically united database of 16S rRNA gene sequences and whole-genome assemblies," *International Journal of Systematic and Evolutionary Microbiology,* vol. 67, no. 5, p. 1613–1617, 30 May 2017.

[4] C. Djemiel, D. Plassard, S. Terrat, O. Crouzet, J. Sauze, S. Mondy, V. Nowak, L. Wingate, J. Ogée and P.-A. Maron, "µgreen-db: a reference database for the 23S rRNA gene of eukaryotic plastids and cyanobacteria," *Scientific Reports volume 10,* 03 April 2020.
